# The Benefits and Cost-Effectiveness of Arteriovenous (AV) Fistula Screening in Haemodialysis Patients

**DOI:** 10.7759/cureus.50185

**Published:** 2023-12-08

**Authors:** Mohamed S M Elshikhawoda, Sohaib Jararaa, Mahmoud Okaz, Mohammed S. M. Houso, Abdelrahman Maraqa, Reyad Abdallah, Eyitomi Terry Kenu, Hussam Khougali Mohamed, Oluwatobi Shekoni, Laszlo Papp

**Affiliations:** 1 Vascular Surgery, Glan Clwyd Hospital, Rhyl, GBR; 2 Surgery, Glan Clwyd Hospital, Rhyl, GBR; 3 General Surgery, Glan Clwyd Hospital, Rhyl, GBR; 4 Vascular Surgery, Royal Oldham Hospital, Oldham, GBR; 5 General and Upper GI Surgery, University Hospital Hairmyres, Glasgow, GBR

**Keywords:** surveillance, stenosis, monitoring, haemodialysis, dysfunctional fistula, arteriovenous fistula

## Abstract

Background

Ultrasound (US) monitoring of arteriovenous fistulas (AVFs) presents contradictory findings. These differences may be attributed to variances in the chosen surveillance strategy, the specific type of fistula being monitored, and the precise technique used for ultrasound scanning. In this study, we are trying to assess the benefits and cost-effectiveness of US scanning of AVF.

Patients and methods

This was a descriptive, retrospective, and observational study. The study sample consisted of patients diagnosed with end-stage renal disease (ESRD) on hemodialysis who had AVF for dialysis either by native vein or using prosthetic grafts. We excluded all the patients whose fistula failed to mature, failed to attend the surveillance scan at six weeks, or had absent records or incomplete data. We retrieved the data of the patients who underwent AVF creation at Glan Clwyd Hospital between April 2020 and April 2023. The data was analysed using statistical software (SPSS) version 21 (IBM Corp., Armonk, NY, USA).

Results

Ninety-eight patients were studied. Stenosis 43.9% (n = 43) was the predominant complication, followed by thrombosis (15.3%; n = 15) while the remaining complications (bleeding, pseudoaneurysm) were less prominent. On the other hand, a total of 37.8% (n = 37) did not experience any complications. Primary patency ranged from 2 to 87 months with a mean of 9 ± 13.2 months SD, and secondary patency ranged from 1 to 24 months with a mean of 1.3 ± 3.9 months SD. The mean cost of a surveillance scan for AVF is 2520 USD, and the mean cost of intervention is 1332 + 1258 USD SD.

Out of all the patients, 52 (53%) underwent intervention to salvage the AVF, 2 (2%) received open surgical intervention, and 50 (51%) underwent endovascular intervention.

Considering the AVF failure to work, 29.6% (n = 29) had fistulas that failed to work, and 70.4% (n = 69) were still working.

Conclusion

Routine duplex scanning in six-month periods to diagnose failing AV fistulas is not cost-effective when compared to diagnosing failing or failed AV fistulas based on clinical symptoms.

## Introduction

Untreated accumulation of toxins and fluid in individuals suffering from end-stage renal disease (ESRD) can be lethal. Therefore, individuals with end-stage renal disease necessitate renal replacement therapy, which can be achieved through either kidney transplantation or dialysis. Approximately 70% of individuals who need dialysis choose to undergo hemodialysis, which is equal to around 20,000 patients annually in the UK [1].

Arteriovenous fistulas (AVFs) are regarded as the most effective method for delivering hemodynamics to patients with end-stage renal disease (ESRD). This is because hemolysis through a central venous catheter (CVC) is linked to a higher occurrence of bloodstream infection, hospitalisation, and expenses [2-8]. The mortality rate for individuals undergoing dialysis through a central venous catheter (CVC) is approximately 40% greater compared to people undergoing dialysis through an arteriovenous fistula (AVF) [9].

The research regarding ultrasound (US) monitoring of arteriovenous fistulas (AVFs) presents contradictory findings. These differences may be attributed to variances in the chosen surveillance strategy, the specific type of fistula being monitored, and the precise technique used for ultrasound scanning.

Ultrasound is a dependable method for identifying fistulas that have fully developed [10,11]. The specific ultrasonic features that define a mature fistula are still a topic of debate [12].

In this study, we are trying to highlight whether the surveillance scan for AVF is cost-effective and has a positive effect on AVF salvage.

## Materials and methods

Study design and population

This was a descriptive, retrospective, and observational study. The study sample consisted of patients diagnosed with end-stage renal disease (ESRD) on hemodialysis who had AVF for dialysis either by native vein or using prosthetic grafts.

We excluded all the patients whose fistula failed to mature, failed to attend the surveillance scan at six weeks, or had absent records or incomplete data.

Data collection

We retrieved the data of the patients who underwent AVF creation at Glan Clwyd Hospital between April 2020 and April 2023.

The clinical evaluation included factors such as the patient's age, gender, comorbidities, smoking, type of fistula, complications, patency, and failure.

Doppler US surveillance

The patient should undergo Doppler ultrasonography (US) to plan the creation of an arteriovenous fistula (AVF) and examine the suitability of the vein and artery for the procedure. Additionally, if there are any anatomical abnormalities, they should be identified. After the AVF is created, Doppler US surveillance should be conducted after six weeks to monitor the progress and detect any complications. In the case of fistula maturation, another scan should be arranged in six months. Vascular scientists with specialised training in arteriovenous fistula surveillance conducted scans using Doppler ultrasound. The study recorded many characteristics on a predetermined study proforma, including the diameter of the radial artery, brachial artery, and axillary artery, as well as the diameter of the outflow vein. Additionally, the presence of flow-limiting stenosis and fistula thrombosis was noted.

Intervention

Following the surveillance scan, if the AVF shows stenosis or other problems like aneurysm or central vein stenosis, a salvage trial was conducted using either open or endovascular treatments. The patients then underwent another scan after six weeks.

Data analysis

The data collected for this study was processed using SPSS 21 software (IBM Corp., Armonk, NY), including data entry, cleaning, and analysis. Descriptive statistics were utilised to present the frequency tables with corresponding percentages Means and standard deviations were also reported. A bivariate analysis was conducted to assess the associations between the outcome variables and other relevant influencing factors. The statistical tests employed were the chi-square test for categorical variables and the t-test for quantitative variables. A significance level of 0.05 or less was considered statistically significant, indicating a substantial relationship between the variables.

Definitions

Primary patency: the interval between access creation and the thrombosis event or the first open surgical or endovascular intervention.

Secondary patency: the interval between access creation and abandonment of the access, including all endovascular and open surgical salvage procedures in between.

Fistula maturation or ready-to-use: any AVF with flow > 400 ml/ minute in the surveillance scan at six weeks.

Fistula failure: AVFs that fail or cease functioning, regardless of salvage intervention.

## Results

We enrolled a total of 98 patients. The majority of the patients were male, accounting for 51% (n = 50), and had a mean age of 67 ± 12.4 years with a standard deviation.

The majority of the patients had a smoking history, accounting for 52% (n = 51), and had additional medical conditions, including hypertension (68.4%) and diabetes (62.2%). Nevertheless, the remaining comorbidities were less common (Table 1).

**Table 1:** Patient demographics and comorbidities DM: Diabetes Mellitus; PVD: Peripheral Vascular Disease

		Number	Percentage
Gender	Male	50	51
	Female	48	49
Ever smoke	Yes	51	52
	No	47	48
DM	yes	61	62.2
	No	37	37.8
Hypertension	Yes	67	68.4
	No	31	31.6
Cardiac diseases	Yes	37	37.8
	No	61	62.2
PVD	Yes	15	15.3
	No	83	84.7
Total		98	100

The preoperative Doppler US for preoperative AVF planning showed an artery diameter ranging from 1.7 to 5.8 mm with a mean of 3.4 ± 1 mm standard deviation (SD) and a vein diameter ranging from 2 to 8 mm with a mean of 3.5 ± 1.3 mm SD.

The most prevalent kind of AVF was the radiocephalic fistula (RCF), which accounted for 39.8% (n = 39) of cases. The brachiocephalic fistula (BCF) followed closely behind, representing 31.6% (n = 31) of cases. The remaining types were less prevalent. Twenty-one (21.4%) patients had AVF before (Table 2).

**Table 2:** Fistula characteristics AVF: Arteriovenous Fistula; RCF: Radiocephalic Fistula; BCF: Brachiocephalic Fistula; BBF: Brachiobasalic Fistula

		Number	Percentage
AVF type	Snuff box	18	18.4
	RCF	39	39.8
	BCF	31	31.6
	Graft	2	2
	BBF	8	8.2
Side	Right	24	24.5
	Left	74	75.5
Previous AVF	Yes	21	21.4
	No	77	78.6
Total		98	100

A total of 95 (96.9%) patients did not demonstrate central vein stenosis while only three (3.1%) patients were diagnosed with this condition.

Stenosis (43.9%; n = 43) was the predominant complication, followed by thrombosis (15.3%; n = 15) while the remaining complications (bleeding, pseudoaneurysm) were less prominent. On the other hand, a total of 37.8% (n = 37) did not experience any complications.

Primary patency ranged from 2 to 87 months with a mean of 9 ± 13.2 months SD, and secondary patency ranged from 1 to 24 months with a mean of 1.3 ± 3.9 months SD. The mean cost of a surveillance scan for AVF is 2520 USD, and the mean cost of intervention is 1332 + 1258 USD SD.

Out of all the patients, 52 (53%) underwent intervention to salvage the AVF, 2 (2%) received open surgical intervention, and 50 (51%) underwent endovascular intervention.

Considering the AVF failure to work, 29.6% (n = 29) had fistulas that failed to work, and 70.4% (n = 69) were still working.

Upon evaluating the factors that could affect or influence fistula failure, we observed that the fistula type and the complications had a significant effect on the failure (P values = 0.045 and 0.000, respectively) (Table 3).

**Table 3:** Factors affecting AVF failure AVF: Arteriovenous Fistula; RCF: Radiocephalic Fistula; BCF: Brachiocephalic Fistula; BBF: Brachiobasalic Fistula

		Fistula failed		P value
		yes	no	
Fistula type	Snuff box	8	10	
	RCF	15	24	
	BCF	3	28	0.045
	Graft	1	1	
	BBF	2	6	
Complications	Bleeding	1	0	
	Pseudoaneurysm	0	2	
	Thrombosis	12	3	0.000
	Stenosis	13	30	
	No complications	3	34	
Total		29	69	

## Discussion

To ensure the optimal functionality of an arteriovenous fistula (AVF), our responsibility is to enhance its longevity through the prevention, timely identification, and immediate management of any problems. To achieve this objective, international guidelines suggest a precise protocol for monitoring vascular access. This involves conducting a physical examination of the arteriovenous fistula (AVF) before each dialysis session. Additionally, surveillance should be carried out on a monthly basis to assess recirculation, venous and arterial pressures, flow volume calculations, and other parameters. These evaluations provide valuable information about the functioning of the AVF [13]. On the other hand, there is adequate evidence that a Doppler ultrasound (DUS) scan surveillance can improve patency [14].

In AVF surveillance, ultrasound can also be employed to investigate the potential factors contributing to vascular access dysfunction. Although the computation of flow volume is relatively straightforward and quick, the systematic examination of an arteriovenous fistula (AVF) using DUS is a complex and time-consuming technique that should only be performed by skilled operators. Hence, this assessment should be employed solely in instances where monitoring techniques have detected irregularities or when complications emerge that impede standard dialysis procedures (such as challenging venipuncture, inadequate blood flow, elevated venous pressure, or prolonged bleeding following the removal of fistula needles).

In this study, stenosis (n = 43) was the predominant complication, followed by thrombosis (n = 15). We had one patient with nonstop bleeding after the removal of the dialysis needle, which needed a ligation, and two patients with pseudoaneurysms that were salvaged surgically, which is aligned with most of the literature [15-17].

The patients with stenosis and thrombosis were offered endovascular intervention such as stenting for central vein stenosis or PTA and/or thrombolysis. However, the vast majority of thrombosis and almost one-third of stenosis patients end up with non-functioning AVF, and secondary patency means not exceeding six months after intervention. Turmel-Rodrigues L et al. concluded that endovascular intervention increased secondary patency for up to three years; this might be due to multiple interventions and the placement of stents to maintain the lumen in cases of restenosis [18].

The mean cost of multiple scans was 2520 USD per patient while the mean intervention cost was 1332 + 1258 USD SD. Hence, despite the multiple scans and interventions, the failure rate was very high among the patients who had endovascular intervention, and the secondary patency was too short. Multiple authors agreed that surveillance and screening improved the patency and survival of AVF. These discrepancies may be due to different study types or interventions [19,20].

Study limitations

The patient count was minimal, with a prevalence of arteriovenous fistula (AVF) cases compared to the other kind, resulting in reduced accuracy.

An inherent constraint of our study was the retrospective collection of data. Therefore, we were only able to examine the data that was already contained within the patient's records.

## Conclusions

There is a general belief among physicians engaged in the establishment and upkeep of vascular accesses for hemodialysis that Doppler ultrasound (DUS) is essential for identifying vessels that are appropriate for creating an arteriovenous fistula (AVF) during preoperative mapping, as well as for promptly detecting any difficulties during surveillance. However, this study demonstrated that there is no discernible advantage or cost-effective utility in utilising ultrasound monitoring of AVF or prolonging their patency. Routine duplex scanning in six-month periods to diagnose failing AV fistulas is not cost-effective when compared to diagnosing failing or failed AV fistulas based on clinical symptoms.

## References

[1] Caskey F, Cullen R (2016). UK Renal Registry 18th Annual Report: introduction. Nephron.

[2] Taylor G, Gravel D, Johnston L, Embil J, Holton D, Paton S (2004). Incidence of bloodstream infection in multicenter inception cohorts of hemodialysis patients. Am J Infect Control.

[3] D'Agata EM, Mount DB, Thayer V, Schaffner W (2000). Hospital-acquired infections among chronic hemodialysis patients. Am J Kidney Dis.

[4] Marr KA, Kong L, Fowler VG, Gopal A, Sexton DJ, Conlon PJ, Corey GR (1998). Incidence and outcome of Staphylococcus aureus bacteremia in hemodialysis patients. Kidney Int.

[5] Rodríguez-Aranda A, Alcazar JM, Sanz F, García-Martín F, Otero JR, Aguado JM, Chaves F (2011). Endoluminal colonization as a risk factor for coagulase-negative staphylococcal catheter-related bloodstream infections in haemodialysis patients. Nephrol Dial Transplant.

[6] Rosenbaum D, MacRae JM, Djurdjev O, Levin A, Werb R, Kiaii M (2006). Surveillance cultures of tunneled cuffed catheter exit sites in chronic hemodialysis patients are of no benefit. Hemodial Int.

[7] McCann M, Moore ZE (2010). Interventions for preventing infectious complications in haemodialysis patients with central venous catheters. Cochrane Database Syst Rev.

[8] Engemann JJ, Friedman JY, Reed SD (2005). Clinical outcomes and costs due to Staphylococcus aureus bacteremia among patients receiving long-term hemodialysis. Infect Control Hosp Epidemiol.

[9] Astor BC, Eustace JA, Powe NR, Klag MJ, Fink NE, Coresh J (2005). Type of vascular access and survival among incident hemodialysis patients: the Choices for Healthy Outcomes in Caring for ESRD (CHOICE) Study. J Am Soc Nephrol.

[10] Ives CL, Akoh JA, George J, Vaughan-Huxley E, Lawson H (2009). Pre-operative vessel mapping and early post-operative surveillance duplex scanning of arteriovenous fistulae. J Vasc Access.

[11] Hindle P, Ritchie J, Griffiths G, Howd A (2011). Use of early scanning in fistula formation for vascular access. J Vasc Access.

[12] Beathard GA, Lok CE, Glickman MH (2018). Definitions and end points for interventional studies for arteriovenous dialysis access. Clin J Am Soc Nephrol.

[13] Schmidli J, Widmer MK, Basile C (2018). Editor's choice-vascular access: 2018 clinical practice guidelines of the European Society for Vascular Surgery (ESVS). Eur J Vasc Endovasc Surg.

[14] Lok CE, Huber TS, Lee T (2020). KDOQI clinical practice guideline for vascular access: 2019 update. Am J Kidney Dis.

[15] Stolic R (2013). Most important chronic complications of arteriovenous fistulas for hemodialysis. Med Princ Pract.

[16] Vajdič Trampuž B, Arnol M, Gubenšek J, Ponikvar R, Buturović Ponikvar J (2021). A national cohort study on hemodialysis arteriovenous fistulas after kidney transplantation - long-term patency, use and complications. BMC Nephrol.

[17] Fokou M, Teyang A, Ashuntantang G, Kaze F, Eyenga VC, Chichom Mefire A, Angwafo F 3rd (2012). Complications of arteriovenous fistula for hemodialysis: an 8-year study. Ann Vasc Surg.

[18] Turmel-Rodrigues L, Pengloan J, Blanchier D, Abaza M, Birmelé B, Haillot O, Blanchard D (1993). Insufficient dialysis shunts: improved long-term patency rates with close hemodynamic monitoring, repeated percutaneous balloon angioplasty, and stent placement. Radiology.

[19] Tessitore N, Bedogna V, Poli A (2008). Adding access blood flow surveillance to clinical monitoring reduces thrombosis rates and costs, and improves fistula patency in the short term: a controlled cohort study. Nephrol Dial Transplant.

[20] Cayco AV, Abu-Alfa AK, Mahnensmith RL, Perazella MA (1998). Reduction in arteriovenous graft impairment: results of a vascular access surveillance protocol. Am J Kidney Dis.

